# Onset of morning activity in bumblebee foragers under natural low light conditions

**DOI:** 10.1002/ece3.7506

**Published:** 2021-05-01

**Authors:** Katie Hall, Théo Robert, Kevin J. Gaston, Natalie Hempel de Ibarra

**Affiliations:** ^1^ Centre for Research in Animal Behaviour, Psychology University of Exeter Exeter UK; ^2^ Environment and Sustainability Institute University of Exeter Penryn UK; ^3^ Present address: Centre for Behaviour and Evolution, Biosciences Institute Newcastle University Newcastle upon Tyne UK

**Keywords:** bees, experience, foraging, learning, navigation, vision

## Abstract

Foraging on flowers in low light at dusk and dawn comes at an additional cost for insect pollinators with diurnal vision. Nevertheless, some species are known to be frequently active at these times. To explore how early and under which light levels colonies of bumblebees, *Bombus terrestris,* initiate their foraging activity, we tracked foragers of different body sizes using RFID over 5 consecutive days during warm periods of the flowering season. Bees that left the colony at lower light levels and earlier in the day were larger in size. This result extends the evidence for alloethism in bumblebees and shows that foragers differ in their task specialization depending on body size. By leaving the colony earlier to find and exploit flowers in low light, larger‐sized foragers are aided by their more sensitive eyes and can effectively increase their contributions to the colony's food influx. The decision to leave the colony early seems to be further facilitated by knowledge about profitable food resources in specific locations. We observed that experience accrued over many foraging flights determined whether a bee started foraging under lower light levels and earlier in the morning. Larger‐sized bees were not more experienced than smaller‐sized bees, confirming earlier observations of wide size ranges among active foragers. Overall, we found that most foragers left at higher light levels when they could see well and fly faster. Nevertheless, a small proportion of foragers left the colony shortly after the onset of dawn when light levels were below 10 lux. Our observations suggest that bumblebee colonies have the potential to balance the benefits of deploying large‐sized or experienced foragers during dawn against the risks and costs of foraging under low light by regulating the onset of their activity at different stages of the colony's life cycle and in changing environmental conditions.

## Graphical abstract

It is widely believed but little evidenced that some diurnal pollinators, such as bumblebees, start foraging very early on flowers to avoid competition. Recording with RFID, we find that despite their high visual sensitivity very few foragers leave at the earliest opportunity in very low light in the mornings of warm days in spring and summer. We further show evidence for alloethism, but extend it by finding evidence for foraging specialisation in larger bees and a role of experience. Both factors enable the colony to adaptively deploy foragers in the early morning when the environment changes or their needs in different stages of the colony's life cycle.

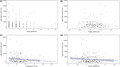

## 1 INTRODUCTION

Foraging on flowers early in the morning is a possibility for diurnal pollinators to reduce predation and competition for floral resources (Kelber et al., 2006; Wcislo et al., 2004). Some flowers open during dawn or secrete nectar before the majority of diurnal insect pollinators becomes active (Ewusie & Quaye, 1977; Oltmanns, 1895; Pacini & Nepi, 2007; van Doorn & van Meeteren, 2003). Accessing these floral resources early in the day can be particularly beneficial for bees because it can increase the influx of nectar and pollen to provision their nest or colony (Gottlieb et al., 2005; Wcislo & Tierney, 2009). However, at this time of day light levels are low, limiting the performance of the apposition compound eyes of bees and most other diurnal insects (Warrant et al., 2008). Only few bee species have evolved neural and optical adaptations that enable them to see, navigate, and forage in very dim light, or even at night under moonlight and starlight, such as *Megalopta genalis* and *Xylocopa tranquebarica* (Greiner et al., 2004; Somanathan et al., 2009; Theobald et al., 2006). Similar capacities were suggested for miniature‐sized tropical stingless bees that have been observed flying around the nest at dawn and dusk; however, it remains unclear whether they rely on vision alone under these light conditions (Koethe et al., 2020; Schmidt et al., 2003; Streinzer et al., 2016). More commonly, larger‐sized bee species, such as diurnal carpenter bees, the giant honeybee and bumblebees, can be observed visiting flowers during dusk and/or dawn as facultative dim‐light foragers. Light levels are still low at these times of day, and activity comes at a cost to their navigational and foraging efficiency (Somanathan et al., 2009). For example, bumblebees, *Bombus terrestris*, fly and land under low‐light illumination more slowly than in bright light, and their flight trajectories are more circuitous (Kapustjanskij et al., 2007; Reber et al., 2015, 2016). To understand which traits enable low‐light foraging, we conducted a systematic study tracking the onset of forager activity throughout the morning in bumblebee colonies and measuring the size and level of experience of the foraging individuals.

Bumblebees are generalist foragers and exhibit individual preferences and flower constancy for diverse pollen and nectar sources (reviewed by Goulson, 1999; Heinrich, 1983; Nicholls & Hempel de Ibarra, 2017). They vary in their body size; for example, in *Bombus terrestris,* thorax sizes range from 2.5 to 6.9 mm (Goulson et al., 2002), which is similar to the sizes of other social and solitary bee species (Greenleaf et al., 2007). Foragers tend to be larger in size compared to their nest mates that tend to the colony, a size‐dependent form of behavioral specialization termed alloethism (Goulson et al., 2002; Jandt & Dornhaus, 2014; Yerushalmi et al., 2006). Larger bumblebee workers are more costly to rear (Kerr et al., 2019), but they outperform smaller‐sized workers in a number of behaviors, such as increased nectar foraging rates (Peat et al., 2005; Spaethe & Weidenmüller, 2002), faster flight speed inside flower patches (Pyke, 1978), faster thermoregulation (Heinrich & Heinrich, 1983), and faster ingestion of nectar (Harder, 1983). They also invest more in learning about flowers (Frasnelli et al., 2021).

However, small bees are not insignificant to the colony. When exposed to starvation, they have been shown to live significantly longer than larger bees, which might be vital for colony survival during times when nectar supplies are scarce (Couvillon & Dornhaus, 2010). Both small and large bumblebees contribute in different ways to the functioning and development of a colony.

We measured at which light levels foragers of different body sizes departed from the nest in the morning. Based on the aforementioned studies, we expected that bees would frequently depart from their nest under low light conditions. We predicted that those leaving in dim light would be larger in size. Eye size scales with body size in bumblebees, increasing the number and size of facets, which makes larger eyes more sensitive under low light conditions (Spaethe & Chittka, 2003; Taylor et al., 2019). They cannot fly when it is completely dark. At light levels above 3 lux, they are able to control their flight and landing movements effectively (Reber et al., 2015, 2016). At even lower light levels, a flight reflex can be elicited in larger‐sized foragers (Kapustjanskij et al., 2007), which possibly contributes to better resilience when foraging out in the field.

We also tracked the foragers' individual foraging experience. Bumblebees develop their initial foraging trajectories within few flights but also develop multidestination routes with further flight experience (Osborne et al., 2013; Woodgate et al., 2017). Bumblebee foragers with more experience have been shown to travel over larger distances and to visit patches of flowers along foraging routes which they establish and learn individually to maximize their foraging efficiency (Lihoreau et al., 2010; Ohashi et al., 2008; Thomson, 1996). Thus, if experienced bees adhere to their established travel routes under low light levels, they could reach foraging patches safely and benefit from an early start in the morning.

## 2 MATERIALS AND METHODS

### 2.1 Subjects and study area

Seventeen colonies of the buff‐tailed bumblebee, *Bombus terrestris* L., were supplied from the commercial breeder Koppert Biological Systems. The study was conducted in two different locations in England, the UK (first location *N* = 9 colonies, May–June 2018, July–September 2019; second location *N* = 8 colonies, July–September 2018, May–July 2019).

### 2.2 Experimental setup

In each location, only one colony was tested at any time. The colony was inside a smaller box and placed inside a dark, large wooden box (Figure S1). The wooden box was placed inside a room, approximately 50 cm from a window that was blocked off and that only had aperture for the exit of a black tunnel. Through this exit, tunnel bees could reach the outdoor environment and also return to the colony. Connected to the exit tunnel were vertical and horizontal transparent tunnels inside the wooden box. These tunnels had shutters and a dorsal opening to divert and capture bees for marking during the initial phase of the experiment. The outdoor environment was a mix of urban gardens (first location) or a rural agricultural landscape with hedges (second location).

Two black RFID reader blocks with a bumblebee‐sized aperture (iID2000, 2k6 HEAD; Microsensys GmbH) were placed between two of the connecting tunnels, near the outdoor exit (7 cm). Bees had to cross through these readers to reach the outdoor exit or return to the colony. We could record the direction of movement and the crossing frequency for each individual. The bees were exposed to outdoor light once they passed through the RFID readers and reached the exit of the tunnel. The distance between the colony exit and outdoor exit was 64 cm, with 17 cm the vertical direction in an L‐shaped drop. Two waterproof temperature and light data loggers (6 × 3 × 2 cm, A‐002–64 HOBO^®^) were used in the experiment, one located outside at the exit of the colony and the other on top of the hive inside the wooden box to confirm that there were no unplanned exposures to light or unusual temperature fluctuations inside of it. In 2018, light and temperature were automatically measured every 15 min and in 2019 every 5 min.

### 2.3 Experimental procedures

Each experimental cycle lasted approximately 2 weeks. It was replicated for each of the 17 naïve colonies. Each colony contained many foragers and arrived individually from the commercial breeder shortly before the start of the experimental cycle. We assume that colonies were in a comparable stage in their life cycle. Upon arrival, a colony was left to settle for 5 days prior to the start of the experiment. Three days after arrival, the bag with sugar syrup that is supplied by the commercial breeder was removed.

#### 2.3.1 Phase 1

For two or 3 days, forager bees were released from the colony to forage outside. The doors in the tunnels were operated selectively to allow bees to leave and return to the colony between 10:00 and 16:00 (BST/GMT+1). Any unmarked bee returning with pollen was diverted into the vertical tunnel, gently caught in a marking tube and marked with a RFID tag (mic3^®^‐TAG 64‐bit RO, iID2000, 13.56 MHz system, Microsensys GmbH) that was attached to the thorax with an epoxy glue (Araldite). After the glue had dried, each bee was released back into the tunnel from where it could enter the colony. Marked bees emerging from the colony were allowed to continue foraging. When returning, any marked bee was also diverted through the vertical tunnel and gently caught in a tube. Its ID number was read out with a manual iID® PEN reader (PENmini USB, 13.56 MHz system; Microsensys GmbH) and then allowed to return to the colony. Flights were tallied for each marked bee, and bees accumulated between 1 and 14 flights during *Phase 1*. By the end of the second or third day, when a sufficiently large number of bees had been marked (on average 50 per colony, min/max 24–87), the colony's exit hole was closed with a shutter while the entry hole remained open to allow bees to return. The exit hole was opened again later on the same day, 30 min after sunset when it was too dark outside for bees to resume foraging. Foragers could then leave the colony the next morning and throughout the following 5 days in *Phase 2* of the experiment.

#### 2.3.2 Phase 2

Over the next 5 days, bees left and returned to the colony without any restrictions and interferences. The activity of bees was logged continuously over 24 hr with the RFID apparatus. In the morning of the sixth day, the exit hole was closed while keeping the entry hole open. We waited for a small number of bees that returned to the colony during the next 3–5 days. The RFID loggers continued to record during these days, and the colony was provisioned with sugar syrup and pollen.

After *Phase 2* ended, the colony was frozen to extract bees for size measurements. The ID number of the tagged bees was determined by using the iID^®^ PEN reader. Thorax size (intertegular span) was determined with digital callipers (Louisware, resolution: 0.01 mm) by averaging three measurements per bee.

### 2.4 Data treatment and statistical analysis

Raw data comprised the ID number of the bee, time stamp, the date, and corresponding reader ID. Sequences and durations of foraging trips were extracted using custom‐written code in the software package R statistical interface v. 1.1.453. The measurement of light and outdoor temperature nearest in time to a bee's exit was recorded. Statistical analysis for all tests was completed in the R statistical interface RStudio v. 1.2.5019. All models were computed using the lme4 package in R and underwent validation to check that the assumptions of the model were satisfied (Bates et al., 2015). Plots were made using the ggplot2 package in R (Wickham, 2016).

Across the 17 colonies, 993 individual bees were tagged in *Phase 1* but only 520 were recorded during *Phase 2*. Some tagged bees were found in the colony at the end of the experiment, and others were lost during *Phase 1*. Out of a total of 19,184 completed trips that were recorded during *Phase 2*, 81% lasted longer than 6 min. In previous studies, short trips, 5–10 min, were considered not to be foraging flights (Ings et al., 2006; Ings et al., 2005; Spaethe & Weidenmüller, 2002). A visual inspection of the flight distribution histogram showed that a majority of flights were clearly longer than 6 min. At the other extreme, there were a number of readings that resulted in very short durations with a prominent peak around 1 min of duration. Subsequent inspections showed that these short recordings registered a different group of bees that did not leave the nest during the early or late morning to conduct longer flights. In agreement with the literature, we therefore did not consider flights of less than 6 min of duration as foraging flights in the present study.

We analyzed the 7,376 exits that took place between 03:00 and midday, 12:00 (BST/GTM+1), of which 84% were foraging flights (Figure S2a,b). Nautical dawn times were extracted from the maptools packages in R and were 03:05 and 05:34 in the evening (BST/GTM+1) (Bivand and Lewin‐Koh, 2020). Only bees that completed at least one foraging flight on at least two out of the 5 days in *Phase 2* were included in the data and considered to be active foragers. We focused the analysis on the first and last foraging day of a bee in *Phase 2*, because some bees did not forage every day or went missing in the field. Bumblebees are known sometimes to stay in the field overnight and return to the colony the next day. Because it is unknown whether and when a forager has foraged when it returns in the morning from such overnight stay in the field, we did not include departures of bees where a bee had spent the night preceding their first or their last foraging day, respectively, outside of the colony in *Phase 2*.

Foraging experience prior to a bee's first foraging day was determined from the tally of flights for each bee in *Phase 1* (henceforth termed early foraging experience). Accordingly, the experience a bee had accrued prior to its last foraging day is represented by both the flights in *Phase 1* and the foraging flights on the preceding foraging days in *Phase 2*.

To establish whether foraging experience and/or thorax width predicted at which light level bees initiated foraging on their first or last foraging day during *Phase 2*, respectively, we used a generalized linear mixed model with a gamma error structure and log link function. The main effects in the models were early foraging experience (for the first foraging day) or accrued experience (for the last foraging day), respectively, and thorax width. Colony was nested within season as a random factor. In order to explore whether circadian rhythms might differ between bees of different size and experience, a generalized linear model with a Gaussian error structure and inverse link function was used to test the relationship between time since nautical dawn. The factors were the same as described above.

## 3 RESULTS

### 3.1 First foraging day

**FIGURE 1:**
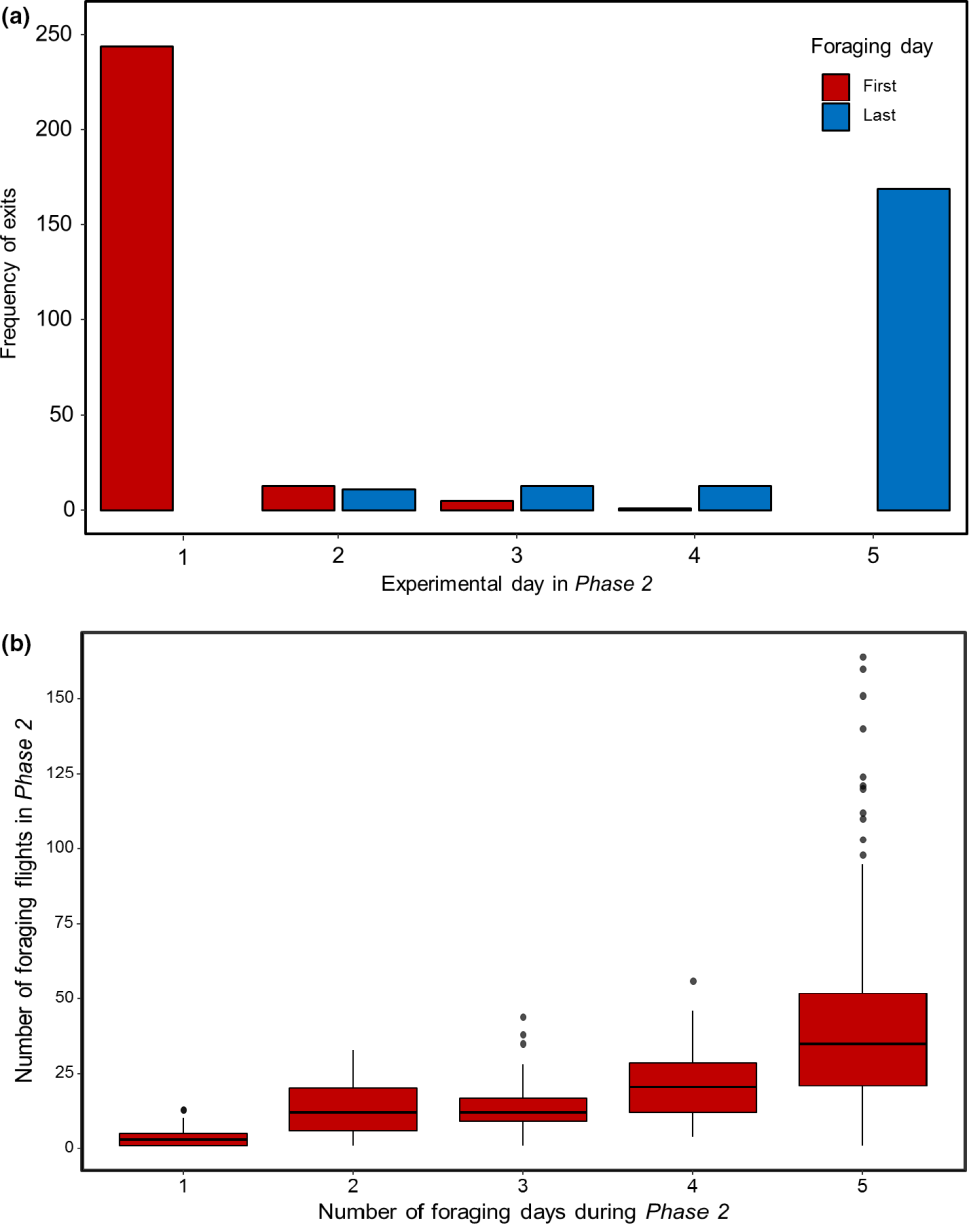
Foraging activity in *Phase 2*. (a) Frequency of the initial flights on the first foraging day (red bars, *n* = 263 bees) and the initial flights on the last foraging day (blue bars, *n* = 206 bees) during five experimental days of *Phase 2*. (b) Bees completed more foraging flights during *Phase 2* when they foraged on more days (*n* = 511 bees)

**FIGURE 2:**
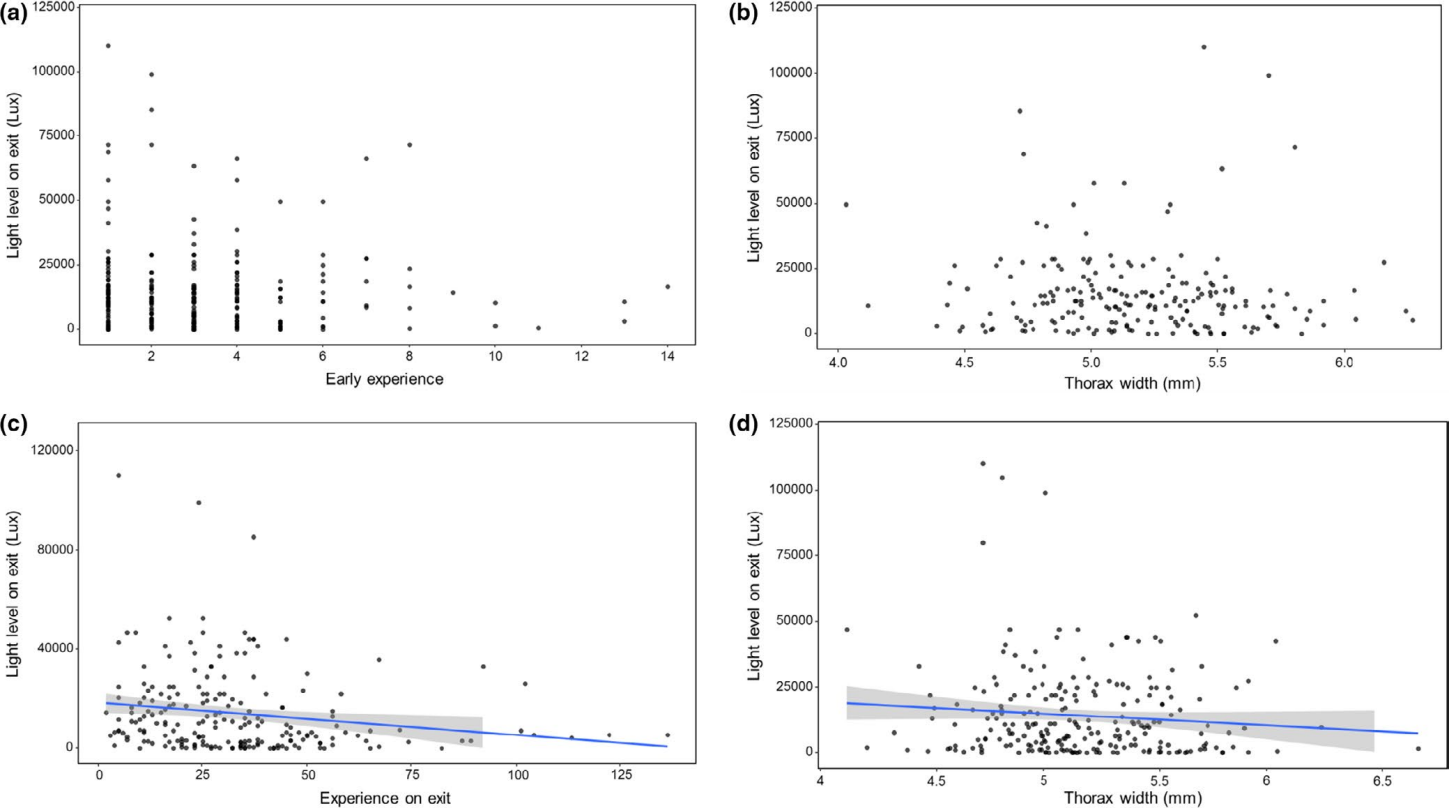
Light levels at the tunnel exit when bees left the colony for their initial foraging flight. Shown are the light levels on the first foraging day against a bee's (a) early experience (*n* = 263 bees) and (b) body size (*n* = 202 bees). A significant relationship was found between light levels on the last foraging day and (c) the accrued experience (*n* = 206 bees) or (d) body size (*n* = 149 bees)

Most bees were active for at least two different days during *Phase 2*. For most of them, the first foraging day was either the first (92.78%) or second experimental day (4.94%) of *Phase 2* (Figure 1a). Early experience in *Phase 1* (Mean = 3.068, IQR = 3) did not predict at which light levels a bee would initiate foraging on its first foraging day (GZLM, *t* = −0.619, *df* = 196, *p* = 0.536, Figure 2a). There was no relationship between a bee's thorax width and the light level at which the initial flight on its first foraging day began (GZLM, *t* = −1.548, *df* = 196, *p* = 0.1220, Figure 2b).

**FIGURE 3:**
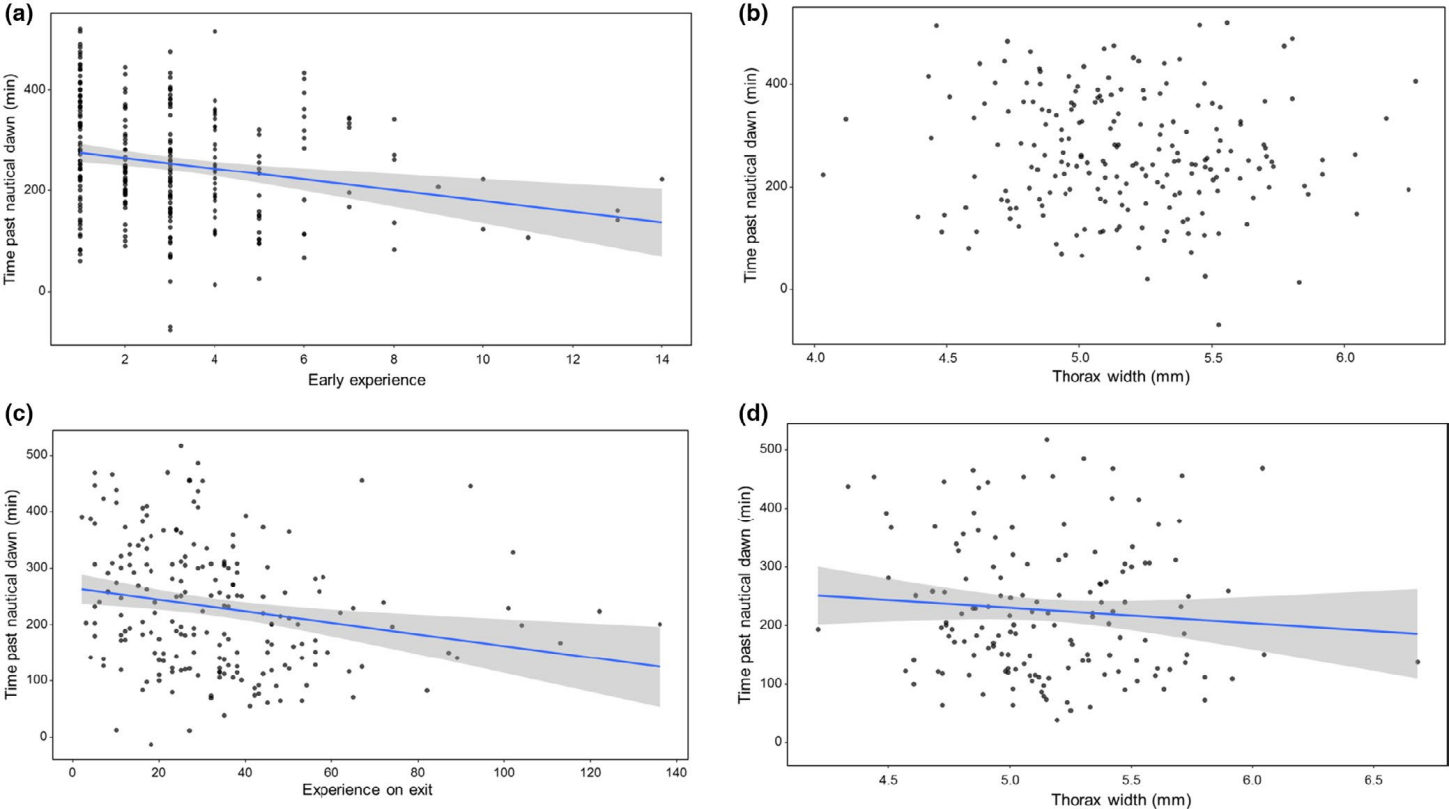
Time relative to nautical dawn at which bees left the colony for their initial foraging flight. Both (a) early experience (*n* = 263 bees) and (c) accrued experience (*n* = 206) determine how early bees left the colony. A relationship between time relative to nautical dawn and the bees' body size is not apparent (b) on the first foraging day (*n* = 202 bees) but is found (d) for the last foraging day (*n* = 149 bees)

Given that foragers display rhythmic activity (Yerushalmi et al., 2006), we also analyzed when after the onset of dawn a bee would leave the colony for foraging. There was a significant relationship between the early experience of a bee and the time after nautical dawn when a bee left (GZLM, *t* = 3.953, *df* = 196, *p* < 0.001) (Figure 3a); however, a larger thorax width did not relate to an earlier onset of a bee's activity relative to dawn (GZLM, *t* = −0.340, *df* = 196, *p* = 0.733) (Figure 3b).

### 3.2 Last foraging day

Across all bees that could be recorded in Phase 2, the number of foraging flights they completed was positively correlated with the number of days a bee went out during *Phase 2*, as predicted (*r*(437) = 0.60, *p* < 0.001) (Figure 1b). Larger bees did not perform significantly more foraging flights compared with smaller bees (GZLM, *t* = 1.194, *df* = 250, *p* = 0.234) (Figure S3). The last foraging day of bees that were active for at least 2 days in *Phase 2* was the fourth (6.31%) or fifth experimental day (82.04%) of *Phase 2* (Figure 1a). By their last foraging day, bees had accrued more experience (mean = 33.01, IQR = 26), as expected (Figure 1b). There was no significant difference between light levels at which a bee initiated foraging on its first and last foraging day (Wilcoxon, *z* = 0.1268, *p* = 0.1120) (Figure S4a) nor between time past nautical dawn at which a bee initiated foraging on its first and last foraging day (Wilcoxon, *z* = 0.0541, *p* = 0.4976) (Figure S4b). This suggests that exposure to full daylight did not simply shift the onset of foraging activity in the observed cohorts to earlier hours and dimmer light.

In contrast to the first foraging day, both the bee's accrued experience (GZLM, *t* = −2.894, *df* = 143, *p* < 0.001, Figure 2c) and body size (GZLM, *t* = −2.256, *df* = 143, *p* = 0.0241, Figure 2d) predicted whether a bee would leave the colony at lower light levels on their last foraging day. There was also a significant relationship between the experience of a bee (GZLM, *t* = 2.823, *df* = 143, *p* < 0.001, Figure 3c) or its size (GZLM, *t* = 3.515, *df* = 143, *p* < 0.001, Figure 3d) and the time after nautical dawn when a bee left the colony.

### 3.3 Early exits observed at the lowest light levels

In the earliest hours, some foragers (*n* = 9) left the colony on either their first or last foraging day when light levels were below 10 lux. While exiting, they crossed the reader only once and returned after staying out for a long time. This suggests that they were out foraging during this time. The sizes of these bees ranged from 5.02 to 5.99 mm. This fits with the overall pattern and our prediction, which is an interesting observation.

Given the warm weather periods we selected for these experiments, air temperatures were sufficiently high so that bees were not discouraged from leaving the colony (Figure S5). We conclude that these foragers departed under very low light levels, although we cannot fully exclude the possibility that they waited at the end of the tunnel for some time until it became brighter. We determined how long it took for light levels to increase above 10 lux during these days (median = 45 min, IQR = 20–70). Their median foraging durations were 71.1 min (IQR = 24.1–224.7), so even including some waiting time bees would have had enough time to forage.

Few other foragers left the colony during dim light (*n* = 4) above 10 lux and below 100 lux on their first or last foraging day. The median foraging durations for these bees were 21 min (IQR = 17–120). Given the exponential rate of light increase, it only took a short time (median = 12.5 min, IQR = 9.5–16.75) when light levels rose to over 100 lux, leaving enough time for foraging. Most other bees began foraging at much higher light levels. On their first foraging day, the median light level was 11,022.4 lux (IQR = 3,444.6–17,222.4) when bees left the colony to forage, and on their last foraging day, it was 8,611.3 lux (IQR = 2,583.5–20,666.9).

## 4 DISCUSSION

Early departures of bumblebees from their nest have been reported previously but not investigated in detail (Spaethe & Weidenmüller, 2002). More commonly, reports of sightings during the early hours of the morning refer to activity of bees on flowers. These can be bumblebee workers that feed themselves after staying in the field overnight, or males after dispersal from their natal nest. These early flower visits may take place under different light conditions than the first flights of foragers when they emerge from the nest. Since little is known about the latter, we tracked the activity of individual foragers from mature, queenright colonies using RFID. The weather and flowering conditions were advantageous for the bees; colonies could easily maintain their pollen and nectar stores and were not experiencing slow temperature increases that would prevent them from leaving the nest (Heinrich, 1972a; Lundberg, 1980; Stelzer & Chittka, 2010; Stelzer et al., 2010).

Some individuals exited the colony under low light conditions, below 10 lux, which is still the range of illumination that allows them to fly and land at flowers in a controlled and sustained manner (Reber et al., 2015, 2016). It shows that bumblebee colonies deploy foragers in the lowest possible light conditions during dawn if required. However, in our experiments it was only a small proportion of the colony's active forager cohort that went out at dawn when the visibility of flowers, landmarks, and horizon was poor. This suggests that bees do not always start foraging as soon as they physiologically might be able to, and that various mechanisms regulate the early‐morning onset of foraging keeping costs low for the colony.

This is the most plausible explanation for the low numbers of active foragers in our study that left the colony in low light conditions during the early hours of the morning. Many more bees were observed leaving the nest at higher light levels. The experiments were conducted during the flowering season, in periods of warm weather and in a species‐rich semiurban environment close to wild habitats. Conditions were stable and favorable so that bees could maximize food collection during the day. Those that went out earlier are likely to benefit from early morning flowers (Ewusie & Quaye, 1977; Oltmanns, 1895; Pacini & Nepi, 2007; van Doorn & van Meeteren, 2003) and residual resources leftover from the previous day (Somanathan et al., 2020). When the majority of bees started later during dawn and the morning, light levels were already high enough for taking full advantage of their visual and flight capabilities and selecting among a larger variety of flowers.

Experience with flowers and foraging routes is an important factor that influences how individual foragers maximize their foraging efforts and contribute to a net influx of food to the colony (Heinrich, 1979a; Leadbeater & Florent, 2014; Spaethe & Weidenmüller, 2002). Since we used naïve foragers for which we had an accurate record of the number of foraging trips, we could explore the role of experience as another factor that might determine which bees leave the colony in the early morning. It is reasonable to assume that bees with little experience risk less if they explore flowers and learn new routes later in the day. Indeed, we found that more experienced bees that completed a higher number of foraging flights were more likely to leave the colony earlier in the morning and under lower light conditions. These were mostly bees that foraged regularly during the whole observation period and would have increased their foraging rate and developed efficient foraging routes (Lihoreau et al., 2012; Osborne et al., 2013; Peat & Goulson, 2005; Raine & Chittka, 2007; Woodgate et al., 2017).

We did not determine the age of the foragers. It is currently unknown whether foraging activity changes inexperienced bumblebee foragers as their age progresses. Most likely, experience rather than age determines which bees are better navigators and foragers. For example, bees learn very fast and establish routes within their first few foraging trips (Osborne et al., 2013), and therefore an effect of age would be difficult to detect in a mixed‐age forager cohort. Interestingly, it might be less beneficial for old bees to forage if they suffer from cognitive decline as suggested for honeybees (Behrends et al., 2007). These are certainly interesting questions for future studies.

Besides foraging experience, we found that a larger body size is associated with a bee's propensity to leave the colony in dimmer light or earlier in the morning. This is consistent with previous findings showing that large foragers have larger eyes and therefore lower sensitivity thresholds (Taylor et al., 2019) and show flight reflexes under lower light levels than small bees (Kapustjanskij et al., 2007). Large workers that forage are also more likely to show diurnal rhythmicity than bees that stay inside the nest and are smaller in body size (Yerushalmi et al., 2006). The earliest departures in the morning coincide with lower illumination, and therefore, rhythmic activity could also determine when an individual starts foraging.

It can be beneficial for the colony if large foragers depart earlier and in lower light conditions. With their more sensitive eyes, they are more likely to navigate safely and reach familiar flower patches, and explore new flowers more easily. They can also fly faster between flowers than their smaller‐sized nest mates (Cresswell et al., 2000; Pyke, 1978), and this could be advantageous in low light conditions. Recent work has also shown that larger bumblebees, compared with smaller ones, have a stronger phototactic response (Merling et al., 2020). Nevertheless, colonies do not seem to have a segregated “caste” of specialized foragers with large body sizes. Bees engaging in foraging vary over a wide range of body sizes, which overlaps strongly with the sizes of bees that tend to the nest (Cumber, 1949; Goulson et al., 2002; Jandt & Dornhaus, 2009; Yerushalmi et al., 2006). Furthermoe, the proportion of large‐sized bumblebees does not simply increase linearly as the flowering season progresses and the colony becomes older. It depends on the colony's state that changes during various maturation periods in a colony's life cycle. Growth trends vary as a result of fluctuating levels of brood, workers and food influx (Couvillon, Fitzpatrick, et al., 2010; Pereboom et al., 2003; Sutcliffe & Plowright, 1988; Sutcliffe & Plowright, 1990).

Another consideration is that the rearing of large‐sized workers is costly for the colony (Kerr et al., 2019), even though they contribute considerably to its maintenance. Larger bumblebees can carry heavier loads of nectar and pollen (Goulson et al., 2002; Spaethe & Weidenmüller, 2002). They also diversify the colony's food, exploiting flowers with sophisticated opening mechanisms that are only accessible to large‐sized pollinators (de Luca et al., 2013; Heinrich, 1979b; Peat et al., 2005). They thermoregulate more efficiently, heating up their flight muscles faster when departing from a flower (Bishop & Armbruster, 1999; Heinrich & Heinrich, 1983). Our recordings show that temperatures were within a range in which bumblebees frequently forage (Couvillon, Fitzpatrick, et al., 2010; Couvillon, Jandt, et al., 2010; Heinrich, 1972; Peat & Goulson, 2005). Nevertheless, thermoregulation is likely to be an additional cost incurred by bees that forage very early in the morning, as bees keep their flight muscles at the appropriate temperature during landings on flowers and when moving between them in a patch (Heinrich, 1972b). Thus, relying on both large‐sized foragers and experienced foragers enables the colony to respond adaptively to floral resources that are available early in the morning.

Several mechanisms could influence a forager's decision to emerge from the nest in the morning. Here, we show that individual foragers are active at different times in the early hours of the morning depending on their experience and also body size. What remains to be understood is how their individual circadian rhythms are entrained by natural sunlight during the transition from night to day, and whether this provides the colony with a further mechanism for deploying sufficient numbers of foragers in the early morning hours. Previous research shows that foraging bees show strong rhythmicity in their activity pattern that is entrained by natural light (Yerushalmi et al., 2006, reviewed by Chole et al., 2019). Furthermore, it is possible that the onset of foraging activity could be strongly influenced by the repeatability of early‐morning foraging behavior. A recent study in honeybees found that expression of clock genes was altered widely across the brain when experimentally separating light entrainment and availability of food (Jain & Brockmann, 2018). Understanding how circadian rhythms develop and whether they determine when individual foragers leave the nest when is another interesting topic for future research.

Finally, it is yet unknown whether social activation of foragers inside the colony (Dornhaus & Chittka, 2001; Molet et al., 2008) occurs in the early morning hours in similar ways as during the day when an increase of foraging activity coincides with the most favorable foraging conditions. For instance, in honeybees the decision to dance and recruit nest mates is influenced by the forager's assessment of their own foraging costs (De Marco, 2006; Dyer, 2002; Von Frisch, 1967). Early‐morning foragers could potentially prioritise individual foraging returns over recruitment.

To benefit from the available floral resources and lower levels of competition in the early morning hours, it might be sufficient for the colony to deploy only few, most effective foragers. This could be an adaptive strategy when environmental conditions throughout the day are favorable. But bumblebee colonies may well have the potential to adaptively regulate the onset of their activity in order to respond to changing conditions in the colony and the environment. The thresholds that determine the division of labor between workers and their sensitivity to social cues and colony stores could vary with size, experience, and possibly individual levels of entrained rhythmicity, thus increasing or decreasing their propensity to leave the nest in the early morning. Whilst in the present study, we observed bees from colonies that were in the same stage of maturity, further research could examine the differences in the foraging patterns of colonies in various stages of their life cycle to understand how foraging time is utilised across the morning and day. With variable environmental conditions during the flowering season and during critical stages of colony development, the state of the colony could change, to a degree that the colony would rely much more on the foraging opportunities in the earliest hours of the day.

## CONFLICT OF INTEREST

There are no conflicts of interest to declare.

## AUTHOR CONTRIBUTION

**Katie Hall:** Conceptualization (equal); Data curation (lead); Formal analysis (lead); Funding acquisition (equal); Investigation (lead); Methodology (equal); Project administration (lead); Validation (lead); Visualization (lead); Writing‐original draft (equal); Writing‐review & editing (equal). **Théo Robert:** Methodology (equal); Software (lead); Validation (supporting); Writing‐review & editing (supporting). **Kevin J. Gaston:** Formal analysis (supporting); Funding acquisition (supporting); Methodology (supporting); Supervision (supporting); Writing‐review & editing (supporting). **Natalie Hempel de Ibarra:** Conceptualization (equal); Formal analysis (supporting); Funding acquisition (equal); Investigation (supporting); Methodology (equal); Project administration (supporting); Resources (lead); Software (supporting); Supervision (lead); Validation (supporting); Writing‐original draft (equal); Writing‐review & editing (equal).

## Supporting information

Figure S1‐S5

## Data Availability

The research data supporting this publication are openly available from the University of Exeter's institutional repository ORE at: https://doi.org/10.24378/exe.3183
