# Supplementary material for: Onset of morning activity in bumblebee foragers under natural low light conditions
Source: Ecol Evol. 2021 May 1;11(11):6536–45. doi: 10.1002/ece3.7506 (PMC8207423; doi:10.1002/ece3.7506)
Supplement: Supplementary file 1 — Figure S1‐S5 [file ECE3-11-6536-s001.pdf]

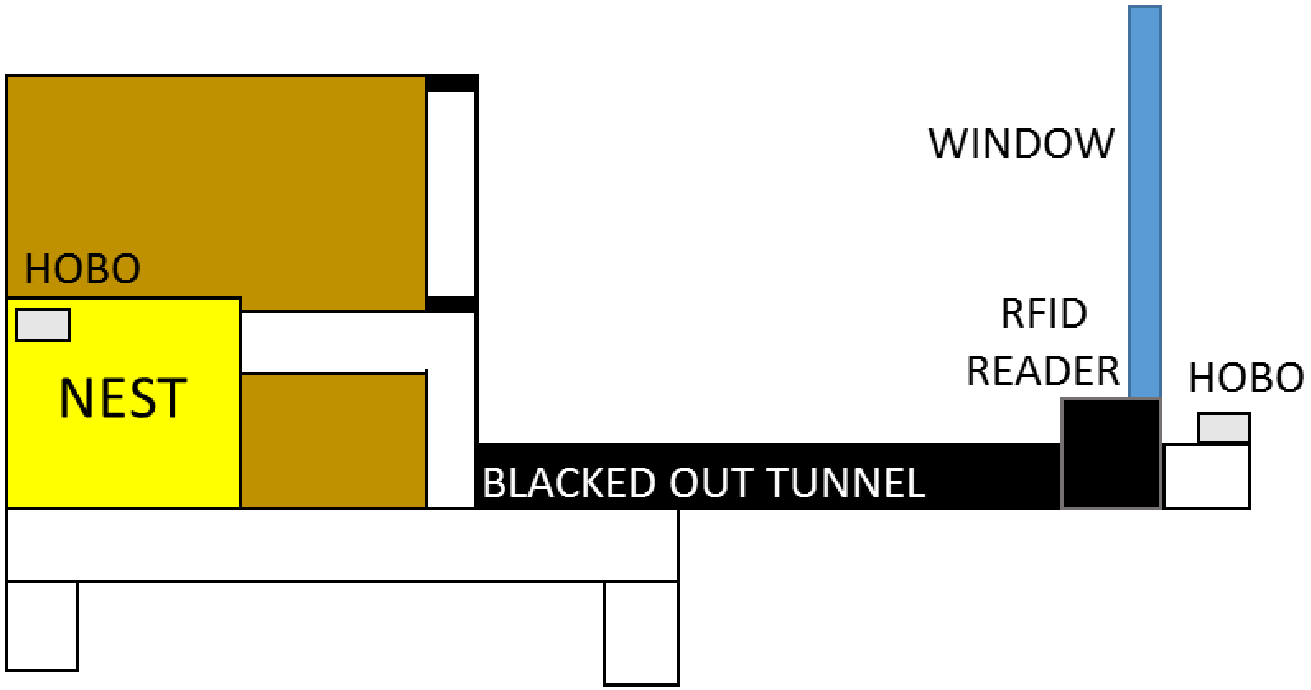

Figure S1. Experimental set up.

The colony in its nest box (yellow) was placed inside a large wooden box with a front door (brown). The wooden box was kept closed during most of *Phase 1* and all of *Phase 2* of the experiment. Tunnels connected the nest exit with the outer environment. Outside the wooden box the tunnels were blacked out. Two black RFID reader blocks with a small hole just large enough to let a bumblebee through (iID2000, 2k6 HEAD; Micro-Sensys GmbH, Erfurt, Germany) were inserted between the tunnels, near the outdoor exit (7 cm). A HOBO device (A-002-64 HOBO®, USA) was installed inside the wooden box to check the light and temperature levels after the end of the experiment. The second HOBO device was installed at the tunnel exit to record ambient light levels and temperature.

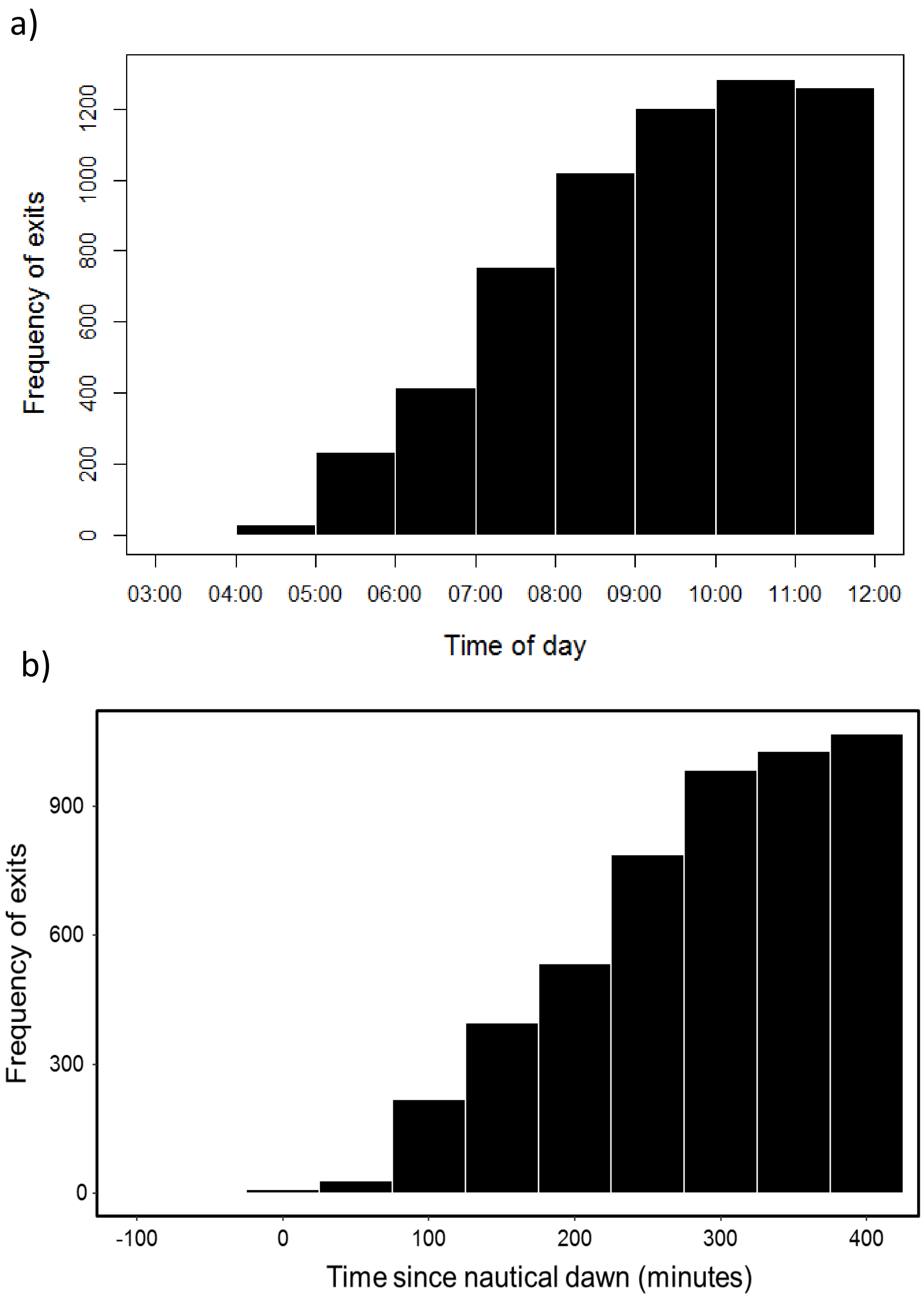

Figure S2. Frequency of foraging flights in *Phase 2*. Recorded number of exits between **(a)** 03:00 and 12:00 noon (N=6208) and **(b)** relative to nautical dawn (N=5598). Nautical dawn fell between 03:05 and 05:34 in the morning (BST/GTM+1) during the experiments.

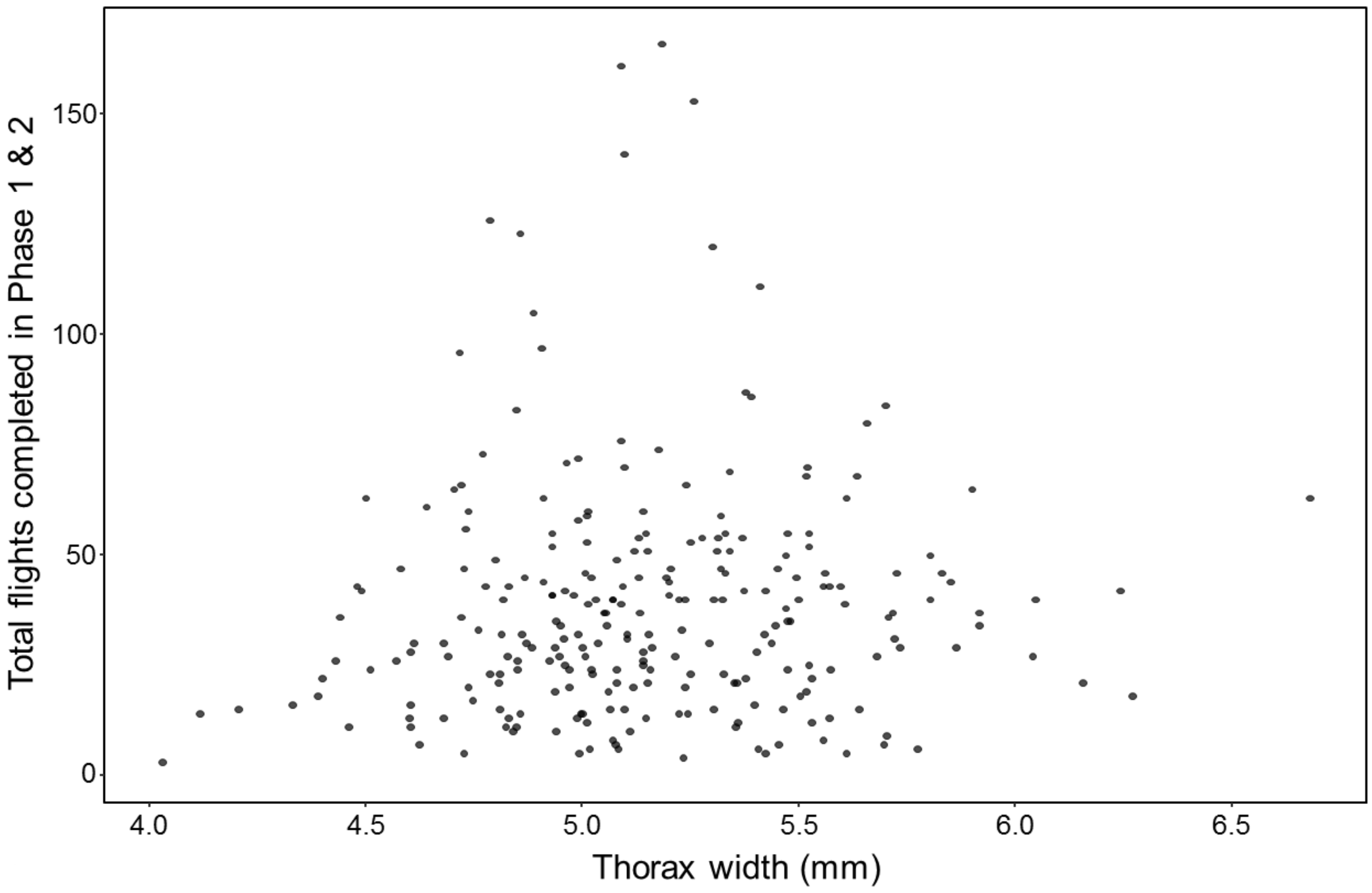

Figure S3. Comparing the amount of foraging completed by bees of different sizes. No association was found between thorax width and the total number of foraging flights (n=230 bees).

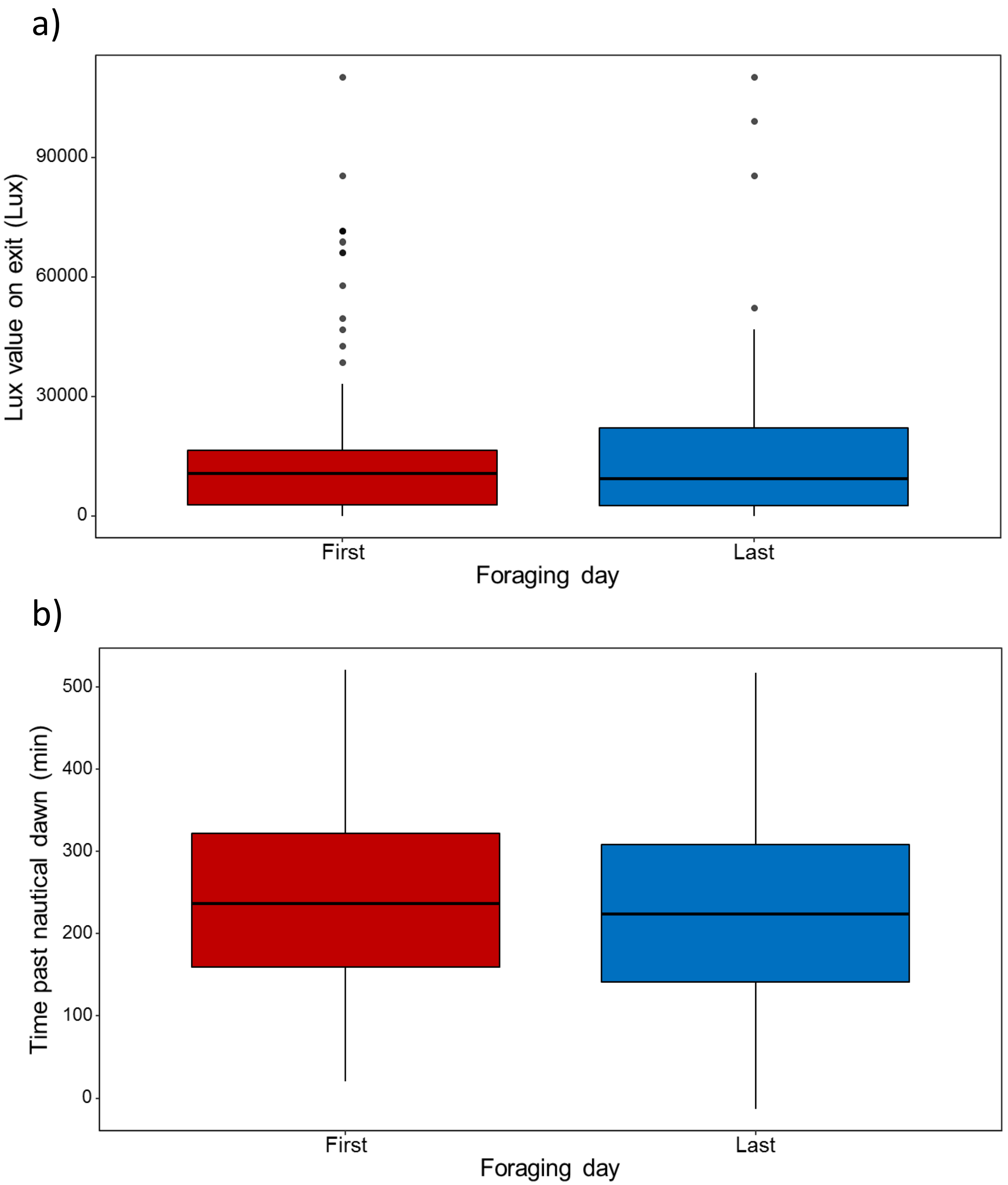

Figure S4. Comparing departure times and light levels on the bees' first and last foraging day. (a) Light levels when bees departed for their initial flight on the first and last foraging day, and **(b)** time relative to nautical dawn (n=157 bees). A paired comparison did not show a significant difference between when a forager initially left the colony on its first and last foraging day against light level **(a,** Wilcoxon, z=0.1268, p = 0.1120**)** and time past nautical dawn **(b,** Wilcoxon, z=0.0541, p = 0.4976**)** (n=157).

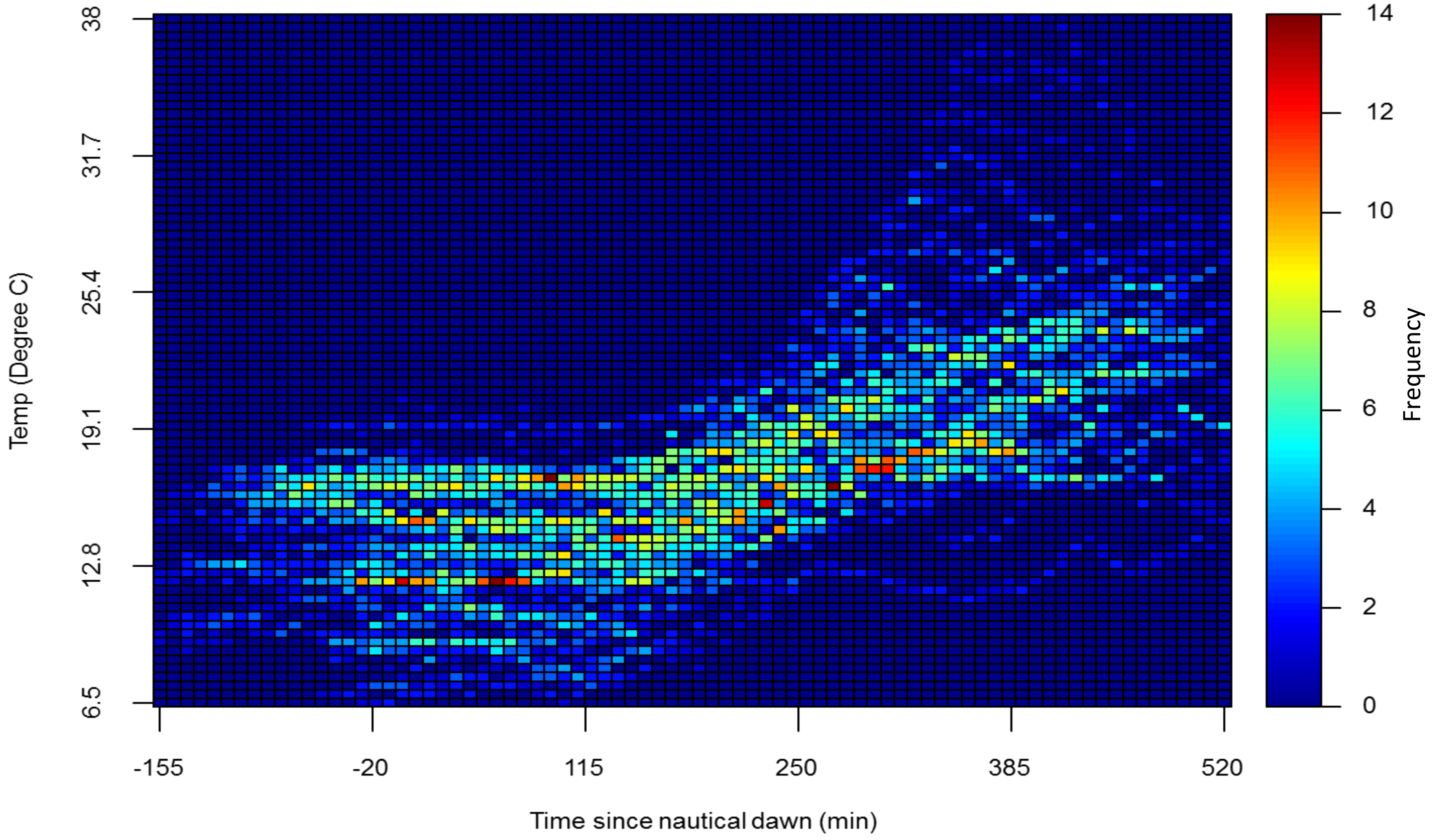

Figure S (5). Temperature conditions during Phase 2 on 85 experimental days between 03:00 and 12:00. Measurements were taken every 15 minutes in 2018 and every 5 minutes in 2019.
